# Gene Therapy for Achromatopsia

**DOI:** 10.3390/ijms25179739

**Published:** 2024-09-09

**Authors:** Megan F. Baxter, Grace A. Borchert

**Affiliations:** 1 Wellcome Trust Centre for Human Genetics, University of Oxford, Oxford OX3 9DU, UK; megan.baxter@lincoln.ox.ac.uk; 2 School of Medicine and Dentistry, Griffith University, Gold Coast 4215, Australia; 3 Nuffield Laboratory of Ophthalmology, Nuffield Department of Clinical Neurosciences, University of Oxford, Oxford OX3 9DU, UK; 4 Oxford Eye Hospital, Oxford University NHS Foundation Trust, Oxford OX3 9DU, UK

**Keywords:** achromatopsia, gene therapy, cone dysfunction

## Abstract

Achromatopsia is the most common cone dysfunction syndrome, affecting 1 in 30,000 people. It is an autosomal recessive disorder with a heterogeneous genetic background with variants reported in *CNGA3*, *CNGB3*, *GNAT2*, *PDE6C*, *PDE6H*, and *ATF6.* Up to 90% of achromatopsia patients harbour mutations in *CNGA3* or *CNB3*, which encode for the alpha and beta subunits of the cone cyclic nucleotide-gated (CNG) channel in cone-specific phototransduction. The condition presents at birth or early infancy with poor visual acuity, nystagmus, photophobia, and colour vision loss in all axes. Multimodal retinal imaging has provided insightful information to characterise achromatopsia patients based on their genotype. There is no FDA-approved treatment for achromatopsia; however, studies have reported several preclinical gene therapies with anatomical and functional improvements reported in vivo. There are currently five gene therapy clinical trials registered for human patients at the phase I/II stage and for *CNGA3* or *CNGB3* causing achromatopsia. This review aims to discuss the genetics of achromatopsia, genotypic and phenotypic correlations in multimodal retinal imaging, and the developments and challenges in gene therapy clinical trials.

## 1. Introduction

Achromatopsia is an autosomal recessive condition affecting an estimated 1 in 30, 000 individuals worldwide [1]. The condition is characterised by reduced visual acuity (typically 20/200), absent or impaired colour discrimination, central scotoma, sensitivity to light, eccentric fixation, and pendular nystagmus [2]. Diagnosis involves clinical evaluation, multimodal retinal imaging, electrophysiological assessment, and genetic testing [3]. Achromatopsia is caused by sequence variants in *CNGA3*, *CNGB3*, *GNAT2*, *PDE6H*, *PDE6C*, and *ATF6* [4,5,6,7,8,9,10]. These genes all encode for important components of the cone phototransduction cascade, which involves a series of processes that convert light into electrical signals, with the exception of *ATF6* [3].

Complete achromatopsia affects all three classes of photoreceptors (long-, middle-, and short-wavelength-sensitive photoreceptors) [1]. This contrasts to incomplete achromatopsia, affecting at least one cone subtype, which is less frequent, with less severe visual impairment (between 20/40 and 20/120), residual colour discrimination, and milder or no photoaversion and nystagmus [11].

There is currently no FDA approved therapy for achromatopsia. Management aims to reduce the impact of photoaversion and reduced visual acuity on quality of life [12]. However, several clinical trials for gene therapy are underway. Since achromatopsia is non-progressive, and because there are residual non-functional cone photoreceptors, there is a good therapeutic window of opportunity for gene supplementation [13]. A recent phase I/II clinical trial of AAV8-hCARp.h*CNGB3* gene replacement therapy in adults and children who have *CNGB3*-associated achromatopsia demonstrated acceptable safety and improvements in several efficacy assessments with 6 months follow-up [14]. This review will examine the achromatopsia clinical genotype and phenotypes, genetic causes, and advancements in clinical gene therapy trials.

## 2. Achromatopsia Phenotypic Patterns

There have been distinct multimodal retinal imaging phenotypic features described in achromatopsia according to genotype [15]. More specifically, fundus autofluorescence, optical coherence tomography (OCT), and electrophysiology have been further characterised in cohorts of achromatopsia patients. There are four distinct fundus autofluorescence phenotypes described: (1) normal, (2) central increased signal, (3) central reduced signal, and (4) central area of decreased signal with a hyperautofluorescent ring [15]. OCT in achromatopsia has been subgrouped into five phenotypic patterns: (1) continuous ellipsoid layer, (2) ellipsoid layer disruption, (3) ellipsoid layer absent, (4) presence of a hyporeflective zone, and (5) outer retinal atrophy with RPE loss [16]. In a cohort of achromatopsia patients with significant follow-up, the OCT findings are generally stable [13]. A common OCT finding in achromatopsia is foveal hypoplasia with incursion of inner retinal layers [16]. Meanwhile, electroretinograms (ERGs) demonstrate normal rod function and an absence of cone-mediated components with more distinct phenotypic features highlighted based on genotypes. Genotype correlations with multimodal retinal imaging and electrophysiology are summarised in Table 1.

Achromatopsia has been well studied with adaptive optics scanning laser ophthalmoscopy (AOSLO) [24]. Confocal AOSLO showed ‘dark spaces’ in the cone mosaic, increased cone spacing, and reduced cone density in achromatopsia patients [25]. However, there is substantial variability in the cone mosaic documented across patients with no significant difference between *CNGA3* and *CNGB3* [25]. In comparison, the rarer *GNAT2* genotype has been observed to have a less disrupted photoreceptor mosaic [17]. A recent longitudinal study of parafoveal cone mosaics, performed on 19 patients with genetically confirmed congenital achromatopsia (1 *CNGA3*, 18 *CNGB3*), was presented at the Association for Research in Vision and Ophthalmology (ARVO abstract 2024 #1428). This demonstrated a small but statistically significant cone density reduction over time for each parafoveal location (ARVO 2024 abstract #1428). However, this was also seen in previously reported longitudinal repeatability values for normal retinas, suggesting that average cone loss may not be pathological in nature (ARVO 2024 abstract #1428) [26].

## 3. Genetic Basis of Achromatopsia Management

**Figure 1:**
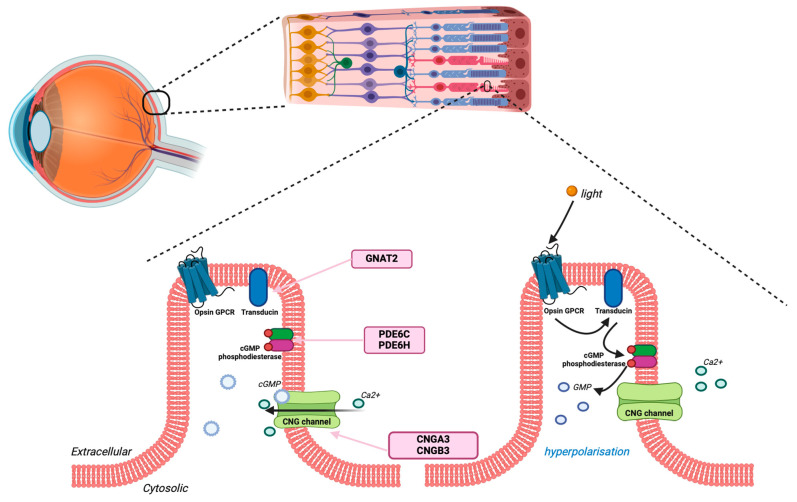
Achromatopsia genes encoding proteins in the phototransduction cascade. Pictorial representation of the cone membrane with resultant intracellular hyperpolarization following light exposure and closure of the CNG channels. Five out of six genes associated with achromatopsia are depicted in pink and are involved in the phototransduction cascade steps indicated by arrows. *CNGA3* and *CNGB3*, the most common achromatopsia genes, encode for two subunits of the cyclic nucleotide-gated channel in the plasma membrane. Meanwhile, *GNAT2* encodes for cone transducin that acts to activate photodiesterase (PDE) to allow for hydrolysis of the second messenger cyclic guanosine monophosphate (cGMP). *ATF6* is not represented in the figure, acting at the endoplasmic reticulum rather than at the plasma membrane. Figure produced using biorender.com (accessed on 15 April 2024).

From a genetics perspective, in more than 90% of cases, a monogenic cause can be identified from one of six key genes within the phototransduction cascade (Figure 1) [27]. *CNGA3* and *CNGB3* genes encode for subunits of the cGMP-gated channel, crucial to the final step of the phototransduction cascade. *GNAT2* encodes for the alpha subunit of the protein transducin in the phototransduction cascade, and *PDE6C* encodes for an enzyme that breaks down cGMP. More than 80% of cases are the result of mutations in either *CNGA3* or *CNGB3* genes or, less commonly, of mutations occurring in the cascade in *GNAT2*, *PDE6C*, *PDE6H*, or transcription factor protein ATF6. In all cases, the inheritance has been identified as autosomal recessive with two abnormal copies of the protein required to cause a phenotypic effect.

The phototransduction cascade allows for the generation of a neural impulse from light through the use of a series of ion currents. In the presence of light, decreased cyclic guanosine momophospahte (cGMP) levels result in the closing of a cyclic nucleotide-gated channel with receptor hyperpolarization and subsequent inhibition of glutamate release [28]. The primary genes that cause achromatopsia are the result of abnormalities in the non-specific cation channel that allows for hyperpolarization. This channel is a tetrameric transmembrane channel consisting of two copies of the *CNGA3* and two copies of the CNGB3 protein. Mutations in *CNGA3* are most commonly missense mutations, and they most commonly occur within a hotspot region in the S4 transmembrane domain [29]. In *CNGB3*, mutations are more commonly nonsense, frameshift, or splice site mutations; the most common mutation is c.1148delC [30]. Both of these genes have shown both homozygous and compound heterozygous inheritance patterns. It is also worth noting that hypomorphic alleles like *CNGB3*/c.1208G>A;p.R403Q with only a partial loss of function have been identified [30]. Given the multitude of different genes, especially across genes involved in single-channel digenic and triallelic inheritance, patterns across *CNGA3* and *CNGB3* have been seen across a very small subset of patients [27,30]. The severity of achromatopsia in these individuals is likely dependent on the individual specific variants, and individuals may demonstrate overlapping features seen in Table 1; however, given the small number of patients, performing genotype–phenotype correlation in these rare inheritance patterns is limited [31].

Variants in *CNGA3* and *CNGB3*, aside from demonstrating mutation hotspots, have also been observed under the effect of both founder effect and population bottlenecks, resulting in highly population-specific variants and variable carrier frequencies. This effect is best demonstrated in the Pingelapese people from the eastern Caroline Islands in Micronesia, with up to 10% of individuals affected by achromatopsia and up to 30% of individuals carrying a single missense mutation in *CNGB3*, c.1306C>T [32]. The identification of this specific variant represents one of the initial and best examples of the use of linkage mapping in the identification of a disease gene. Given the large population prevalence, it allowed for fine mapping and identification of the specific causative locus on chromosome 8q21-22 in 1999 [33,34]. With regards to other specific populations, the most common variant in those of European descent is mutations in *CNGB3*; within those of Asian and Middle Eastern decent, the most common variant is mutations in *CNGA3* [29,35].

Other causative genes identified affect proteins earlier in the phototransduction cascade. During the initial stimulation of the cascade, light stimulates cone opsins, resulting in the activation of a G protein coupled receptor (GPCR). This GPCR is coupled to a cGMP photodiesterase using the protein transducing [28]. The gene *GNAT2* encodes for the alpha protein of the transducin protein. Mutations in *GNAT2* account for approximately 2% of achromatopsia cases [8]. The genes *PDE6C* and *PDE6H* form subunits as part of 3′, 5′ cGMP phosphodiesterase, resulting in the hydrolysis of cGMP and, ultimately, reducing the intracellular concentration of cGMP; these account for approximately 5% of cases [36,37].

Finally, the gene *ATF6* accounts for approximately 2% of cases and is the most recent gene to be identified as causative, encoding a transcription factor regulating the unfolding protein response in the endoplasmic reticulum in response to stress [21]. The protein itself is not directly involved in the cascade, and the exact underlying mechanism remains poorly understood; however, this protein has been shown to have high expression in cones and within all layers of the retina [38].

For the small proportion of patients, less than 10%, with clear phenotypic features of achromatopsia, the cause remains unknown. This could be due to the result of additional non-direct regulatory proteins similar to ATF6 that are yet to be identified. It could also be the result of deep intronic variants in known causative disease genes for which the causative mechanism is yet to be elicited.

## 4. Diagnosis and Testing for Achromatopsia

Despite demonstrating phenotypic heterogeneity, there is a clearly defined phenotypic picture associated with achromatopsia. This allows for the diagnosis to be made clinically following targeted clinical assessment and clinical gestalt [39]. Whist there is often a very clear phenotypic picture, there are other differential diagnoses that should be considered. Individually, each of the symptoms are associated with alternative underlying conditions. For example, congenital nystagmus is often one of the initial symptoms and is associated with numerous structural eye abnormalities [40]. Other eye conditions associated with colour differentiation also need to be considered, including blue-cone monochromatism and tritan and yellow-blue defects. Outside of isolated eye abnormalities, there are also larger syndromic diseases like Alström Syndrome which need to be considered, given very similar early retinal features. Whilst Alström Syndrome is also associated with cardiomyopathy, obesity, type 2 diabetes, renal failure, and sensorineural hearing loss, along with vision issues, initial electroretinography shows severe cone impairment with mild or no rod involvement early in the disease before progression to more severe rod dysfunction as patients become older [41]. Finally, outside of conditions affecting the eye, it is important to exclude a cerebral cause. Cerebral visual impairment is usually associated with other neurological abnormalities secondary to an underlying causative injury, including ischemia or infarction [42].

Genetic testing is therefore important in confirming the diagnosis of achromatopsia. Given the small known subset of disease genes, in the setting of a clear phenotype, a targeted focused panel can be applied to look specifically for exonic changes within known disease genes. If there is ambiguity with regards to the clinical phenotype, there is the potential for more extensive panels to be utilised, including a wider range of genes with known ophthalmic phenotypes [43]. Whilst not usually associated with extraocular manifestations, and not usually associated with other syndromes, there is the potential for more extensive genetic testing to be completed, including whole exome or genome sequencing. Routine, more extensive whole genome testing is associated with not only an increased cost, processing time, and overall increased burden on laboratories but also a high rate of incidental and uninterpretable results. Given that the average patient has four million variants described through WGS, there is huge potential for uninterpretable variant identification [44]. As highlighted by Chen et. Al, predictably, as the number of genes assessed increases, the number of variants of unknown significance (VUS) also increase; hence, limiting unnecessary gene assessment is important [45]. There is also an estimated clinically actionable incidental finding rate in up to 3% of patients [46]. This is of particular significance in the setting of isolated single-organ diseases like achromatopsia, for which any incidental findings are unlikely to be related to the primary disease for which the patient sought assessment. This raises significant ethical issues regarding disclosure and management of future potential disease [47]. As we look to the era of treatment, this may lead to significant overdiagnosis and treatment of disease. Given, however, the clearly defined phenotype, a small list of causative genes, and high penetrance rate, there is often little need for this more extensive testing; this highlights the key principle of the choosing wisely campaign, eliminating unnecessary testing [48].

## 5. Achromatopsia Management

There is currently no treatment available for achromatopsia, so management of the condition is aimed at mitigating the impact on quality of life and genetic counselling. Supportive management includes correcting refraction, low visual aids, and education around the condition. In particular, photoaversion is uncomfortable and can lead to limitations in performing activities of daily living. A patient experience survey reported that 38% of achromatopsia patients described photoaversion as their most significant symptom [49]. Photoaversion experienced by patients with achromatopsia has a significant impact on activities of daily living (ADL) and vision-related quality of life (QoL), which is an important consideration in trials [49]. A qualitative study demonstrated that in 27 patients, 88% reported that light caused significant discomfort, 92% used aids to reduce light, and 56% described that this affected their ADL. It was reported that 96% preferred grey filter rather than red when indoors, and 74% preferred grey filter when outdoors (ARVO abstract #1828).

## 6. Vision Restoration in Pre-Clinical Animal Models of Achromatopsia

**Figure 2:**
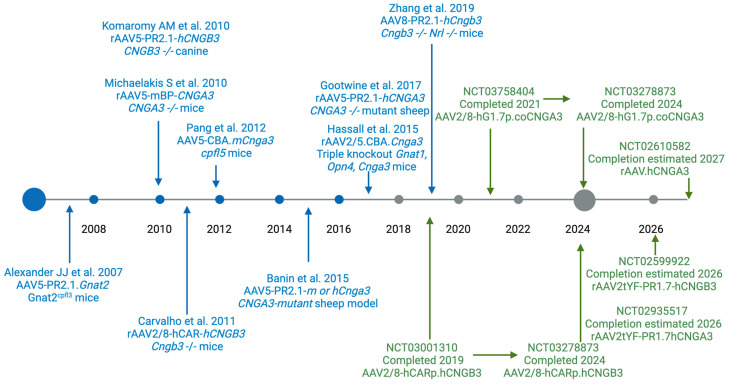
Advancements in achromatopsia from vision restoration in animal models to clinical translation. Restoration of visual function in animal models (blue) over time until 2017 and clinical trials (green) currently registered in ClinicalTrials.gov in 2024 [50,51,52,53,54,55,56,57,58].

Several animal models have been developed to study achromatopsia and test the safety and efficacy of emerging gene therapy (Figure 2). The first restoration of colour vision used *Gnat2* gene therapy on *Gnat^cpfl3^* mice [50]. An AAV5 vector was used with mouse *Gnat2* with the human red/green opsin promoter. Light-adapted ERG responses were restored to 80% of the normal range in vector-treated eyes. This work formed the foundation of the proof of principle for restoring cone function in achromatopsia. Gene therapy rescue was then demonstrated in *CNGA3* −/−, triple-knockout (*Gnat1*, *Opn4*, *Cnga3*), *cpfl5* and *Cngb3* −/− *Nrl* −/− mouse models [51,52,53].

Several dog breeds, such as Alaskan malamute, miniature Australian shepherd, and German shorthaired pointer, are also useful models of achromatopsia. Meanwhile, restoration of visual function was seen in a *CNGB3 −/−* canine, as measured by cone flicker ERG with a follow-up period of 2.5 years [54]. Function was evaluated with an objective behavioural test involving an obstacle-avoidance course. The results of the treated and untreated canine were significantly different at light intensities of >25 lux [54]. 

The therapeutic age window for gene therapy has been investigated in achromatopsia. A mouse model with human *CNGB3* was packaged in an *AAV8* capsid and showed rescue of function, as tested by ERG, at a range of ages, but there was poor response in mice treated at older ages [55]. This suggests that a younger age may be preferable as a therapeutic window.

As a large animal model for human *CNGA3* achromatopsia, Awassi sheep have been used, since they were described as demonstrating congenital day blindness, an autosomal recessive hereditary disease. This included a premature stop codon and missense mutation which was able to be rescued with human PR2.1-*CNGA3* [56]. Restoration was demonstrated by photopic ERG, and there were functional improvements in navigating a maze [56]. *CNGA3* gene augmentation therapy was able to restore vision in a novel causative mutation in Awassi sheep [57].

Preclinical models of nonhuman primate models of *PDE6C* R56Q mutation were tested with AAV5 carrying rhesus *PDE6C* under the control of the cone-specific promotor PR1.7 [59]. It was demonstrated to be safe, although there were variable inflammatory responses. Gene therapy partially restored cone function, as measured on ERG, within a month for the infant NHP models, and this was sustained over the year. Chromatic ERG testing demonstrated restoration of amplitudes in all three cone classes [59].

Animal models have limitations in representing the complexity of the retina and testing gene therapy for achromatopsia but provide important, insightful information for the therapeutic planning strategy for clinical trials. From animal models, it has been suggested that early intervention provides better outcomes compared to older ages. Further, in all studies, the gene therapy was delivered by subretinal injection, and this is likely, since it is challenging to target the outer retina with an intravitreal injection.

## 7. Gene Therapy Clinical Trials for Achromatopsia

There are five registered gene therapy clinical trials on achromatopsia as of June 2024 (Table 2). There are two clinical trials that have been completed, two active trials, and one recruiting. Each gene therapy contains either *CNGB3* or *CNGA3*, based on the reasoning that mutations in these genes affect 80% of achromatopsia patients, and were packaged into an AAV capsid and then delivered by a subretinal injection after vitrectomy. While the primary outcome of phase I/II was safety, the secondary outcomes included visual acuity, electrophysiology, and colour vision.

The phase I/II clinical trial (NCT03001310) tested the safety and efficacy of AAV8-hCARp.*hCNGB3* gene replacement therapy on 23 participants (11 adults and 12 children) with *CNGB3*-associated achromatopsia [14]. The AAV8-hCARp.hCNGB3 demonstrated an acceptable safety profile and was generally well tolerated [14]. There was a trend of more intraocular inflammation with higher doses, which was as expected and anticipated with the surgical route and injection of viral protein. The efficacy was variable, and there was no consistent pattern measured. Positive responses were seen in 6 out of 23 participants for colour vision, 11 out of 20 for photoaversion, and 21 out of 23 in vision-related quality of life questionnaires [14]. One participant reported an improvement in colour discrimination, and two of four children had cone-mediated signals in the visual cortex. Further investigation is needed to determine an appropriate age group for treatment intervention at a time of cortical plasticity and to optimise meaningful, quantitative, and sensitive end points. Long-term follow-up is ongoing for AAV2/8-hCARp.hCNGB3 and AAV2/8-hG1.7p.co*CNGA3* gene therapy (NCT03278873).

A nonrandomised controlled trial evaluated three different doses (1 × 10^10^, 5 × 10^10^, and 1 × 10^11^ total vector genomes per eye) of AAV8.*CNGA3* [60,61]. The treatment was well tolerated and had a good safety profile. Efficacy show some functional benefit, based on cone vision, BCVA, and contrast sensitivity, although the benefit was not statistically significant at 3 years [60].

Preliminary safety and efficacy of AGTC-401 and AGTC-402 in *CNGB3*- and *CNGA3*-related achromatopsia were presented at ARVO. Each had a modified AAV2 capsid with the three surface-exposed Y to F mutations and designated AAV2tYF with a 1.7 kb human M/L opsin promoter. The AGTC-401 included 21 adults and 10 children with *CNGB3* achromatopsia at six different doses. Meanwhile, AGTC-402 was tested in 16 adults and 8 children with *CNGA3* achromatopsia at five different doses. There was some improvement in photosensitivity in *CNGB3* achromatopsia and, to a lesser extent, in *CNGA3* achromatopsia patients. The photoreceptor mosaic integrity was assessed using adaptive optics scanning light ophthalmoscopy (AOSLO) after subretinal injection of AGTC-402 (rAAV2tYF-PR1.7-h*CNGA3*) gene therapy in *CNGA3* achromatopsia. Foveal cone loss seen after subretinal injection was described as a possible result of the surgical procedure or, possibly, a short-term response (ARVO abstract #3289).

## 8. Challenges and Limitations

**Figure 3:**
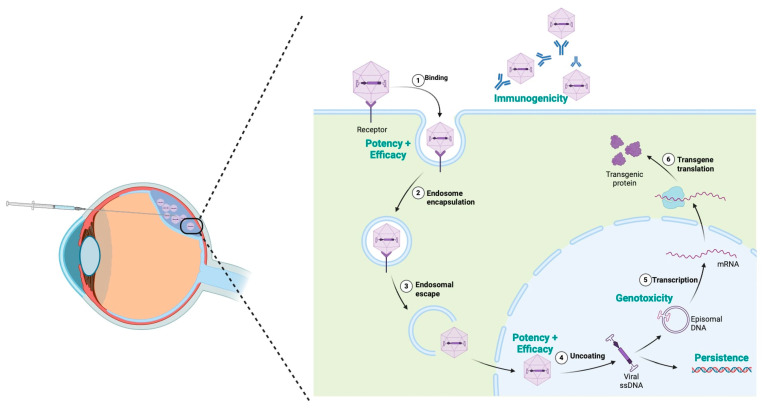
Considerations surrounding the administration of AAV gene therapy. Pictorial depiction of subretinal AAV with associated localised retinal detachment and administration of viral vector. The arrows show the process of cellular uptake of AAV, including uptake into nucleus with transcription and translation of gene of interest. Key concerns, including immunogenicity, potency and efficacy, genotoxicity, and persistence written in teal. Figure produced using biorender.com (accessed on 15 April 2024).

There remain numerous general concerns with gene therapy, along with achromatopsia-specific concerns. For a treatment to be viable, it must meet several criteria, with key areas of gene therapy concern focused around immunogenicity, potency and efficacy, genotoxicity, and persistence, as can be seen in Figure 3 [62]. From an immunogenicity perspective, the eye is often considered to be immune-privileged as the result of a physical barrier, inhibitory microenvironment, and a lack of systemic immune response [63]. This organ isolation is also of significant benefit from a genotoxicity perspective, potentially limiting the potential for systemic complications. While persistence in the epitomal form is critical for the ongoing success of a treatment, if persistence in the genome did occur it would be at the level of somatic rather than germline changes. Each of these are good indicators regarding the potential for success in retinal-based gene therapies [64]. However, these concerns do need to be thoroughly evaluated during early-phase clinical trials [65]. It is also critical that potency and efficacy are assessed, especially with regard to the paediatric patients targeted and intended life-long effect. Thorough investigation and research are associated with significant cost; in addition, development, manufacturing, and administration of gene therapy are costly. These costs remains one of the main limitations in accessing gene therapy [66]. This is compounded with the unfortunate reality seen in many rare disease therapies, despite the huge benefits for affected patents: the small potential patient numbers can often limit pharmaceutical investment in the process [66,67].

For achromatopsia, there are also disease-specific considerations [68]. Achromatopsia is a disease of development and is the result of abnormalities of growth, with the use of the photocascade critical for not only eye development but the establishment of neural networks and maturation of the primary visual cortex. Work by Molz et al. has established that the plasticity of the primary visual cortex is less pronounced than previously reported [69]. Consistent with murine models and early human clinical trials for *CNGA3/B3*, administration in the early paediatric, or even foetal period, may provide the greatest benefit for therapeutic gene therapy trials. There is also a need for pre-therapeutic imaging to better stratify those that may benefit from potential treatment [70]. Further, given pendular nystagmus, there are practical considerations in multimodal retinal imaging, diagnosis, and treatment.

Currently, given the large number of variants, clinical trials have focused on replacement rather than repair of genes. As efficacy of delivery continues to improve, the expression levels will need optimization and the therapeutic range will need to be investigated for safety targets and thresholds [71].

There have been several alternative approaches, apart from gene therapy, for achromatopsia. In patients with achromatopsia caused by *ATF6*, a clinical trial (NCT04041232) has been registered to investigate whether the already approved FDA drug, glycerol phenylbutyrate (PBA), which is a fatty acid compound that facilitates protein folding, can improve retinal function. Meanwhile, a phase I/II prospective single-centre study was performed using a vitreous NT-501 device implant releasing ciliary neurotrophic factor (CNTF) [71]. However, CNTF did not enhance cone function as measured by visual acuity, mesopic increment sensitivity threshold, or photopic ERG [72].

Finally, numerous clinical trials have been carried out which specifically targeted the cyclic nucleotide-gated channel with genes *CNGA3* and *CNGB3*. These studies were of no benefit to the remaining 10% of patients with mutations in genes earlier in the cascade or to the 10% of patients for whom the specific cause was yet to be identified [27]. This highlights the current prioritization of only the ‘common’ disease genes and variants.

## 9. Conclusions and Outlook

In conclusion, this review has highlighted the genetics underlying achromatopsia and the progress paving the way towards promising therapeutics. Advances in multimodal retinal imaging have allowed for in-depth phenotyping to widen the spectrum of achromatopsia. Since there have not been any therapies for achromatopsia that the regulatory agencies have approved, the optimal trial design, inclusion criteria, and the role of modern and spreading vectors should be evaluated, and relevant end points will need to be refined and developed for achromatopsia.

While there is no currently approved treatment for achromatopsia, there have been several promising gene therapy clinical trials. Recently, the use of AAV2tYF-PR1.7-h*CNGA3* and AAV8-h*CNGA3* in achromatopsia have showed preliminary safety and efficacy. Long-term data will be needed to assess the durability of gene therapy treatments. There are lessons to learn from the first gene therapy for inherited retinal dystrophy patients with biallelic *RPE65.* For example, the PERCEIVE study provides important insights into the longer-term safety and efficacy of voretigene neparvovec in a real-world context [73].

It is encouraging to see the advances and developments that are currently being made, in particular, from the perspective of gene therapies. Looking towards the future, we are currently on the precipice of great change. There is a potential to treat this debilitating disease which will truly make a difference to the lives of patients living with achromatopsia.

## Figures and Tables

**Table 1:** Genotype correlations with multimodal retinal imaging patterns in achromatopsia.

Gene	*CNGA3*	*CNGB3*	*GNAT2*	*PDE6C*	*PDE6H*	*ATF6*
**Location**	2q11.2	8q21.3	1p13.3	10q23.33	12p12.3	1q23.3
**CDS length (base pairs)**	2085	2430	1065	2577	252	2013
**Protein encoded**	Encodes alpha-subunit of cyclic nucleotide-gated (CNG) channel	Encodes beta-subunit of cyclic nucleotide-gated (CNG) channel	Encodes G protein alpha-subunit of transducin 2	Encodes alpha catalytic subunit of cone photoreceptor phosphodiesterase	Encodes gamma catalytic subunit of cone photoreceptor phosphodiesterase	Encodes for activating transcription factor 6
**Fundus autofluorescence**	Normal appearance, central increased signal, central decreased signal [2]	Normal appearance, central increased signal, central decreased signal [2]	Typically, normal appearance [17]	Fundus autofluorescence shows decreased central signal with a surrounding hyperautofluorescence [9]	Normal fundus autofluorescence [10]	Variable changes; increased or decreased autofluorescence ring [8]
**OCT**	Foveal hypoplasia in 60–70% of achromatopsia [2]; OCT 50% grade I to III	Foveal hypoplasia in 60–70% of achromatopsia [2]; OCT 50% grade I to III [18]	Foveal hypoplasia was not seen; [19] OCT typically grade I [17]	No foveal hypoplasia; no OCT of grade I and II [9]	No foveal hypoplasia; preserved OCT [10]	Foveal hypoplasia is present in all patients; no reported grades I and II [20]
**Electrophysiology**	ffERG severely reduced cone response with normal/subnormal rod response	ffERG severely reduced cone response with normal/subnormal rod response	ffERG severely reduced cone response; relatively preserved S-cone compared to *CNGA3* and *CNGB3* [17]	Some degree of preserved S-cone similar to *GNAT2*; scotopic ERG can show mild-moderate decrease [17]	Some degree of preserved S-cone similar to *GNAT2* [15]	ffERG severely reduced amplitude of cone response [21]
**AOSLO**	Marked variability [22]	Marked variability [23]	Least disrupted photoreceptor mosaic and reflectivity preserved [17]	Few, if any, cellular residual structures [9]	Not well characterised	Few, if any, remnant cone structures [20]

**Table 2:** Summary of clinical trials for gene therapy in Achromatopsia.

NCT Registration	Phase	Gene	Capsid	Vector	Sponsor	Status	Route
03001310 03278873	I/II	*CNGB3*	AAV5	AAV2/8-hCARp.h*CNGB3*	MeiraGTx/ Janssen	Completed; active	Subretinal
03758404 03278873	I/II	*CNGA3*	AAV5	AAV2/8-hG1.7p.co*CNGA3*	MeiraGTx/ Janssen	Completed; active	Subretinal
02610582	I/II	*CNGA3*	rAAV8	rAAV.h*CNGA3*	RD-CURE	Recruiting	Subretinal
02599922	I/II	*CNGB3*	AAV2tYF	rAAV2tYF-PR1.7-h*CNGB3* (AGTC-401)	AGTC	Active	Subretinal
02935517	I/II	*CNGA3*	AAV2tYF	rAAV2tYF-PR1.7h*CNGA3* (AGTC-402)	AGTC	Active	Subretinal

## Data Availability

Not applicable.
